# Telepsychiatry for mental health triage: A mixed-methods pilot study via a regional health app in Sweden

**DOI:** 10.1177/20552076261429684

**Published:** 2026-03-10

**Authors:** Andreas Påhlsson-Notini, Sigrid Salomonsson, Elin Lindsäter, Sara Arvas, Ulla Forsbeck Olsson, Oscar Norbeck, Maria Bragesjö, Viktor Kaldo, Lina Martinsson

**Affiliations:** 1 Centre for Psychiatry Research, Department of Clinical Neuroscience, 7674 Karolinska Institutet, & Stockholm Health Care Services, Region Stockholm, Sweden; 2 Department of Psychology, Faculty of Health and Life Sciences, 27106 Linnaeus University, Växjö, Sweden

**Keywords:** Telepsychiatry, digital triage, mental health services, implementation, patient experience, feasibility study

## Abstract

Telepsychiatry is increasingly used for mental health triage, but real-world implementation remains underreported, and barriers include technical reliability, patient trust, privacy and coordination across levels of care.

**Objective:**

To describe and evaluate an app- and video-based triage service, Immediate Psychiatry (IP), implemented in Region Stockholm, Sweden, focusing on service use, patient characteristics, acceptability, feasibility and team experiences.

**Methods:**

Adults (≥18) registered with participating primary care centres accessed IP via the regional health app, completed a digital brief screening questionnaire, and had a 45-min video consultation with a mental health professional. A mixed-methods design was employed combining medical-record abstractions, a patient-satisfaction survey, interviews with the IP team and a clinician acceptability questionnaire administered to primary care clinicians. Quantitative data were analysed descriptively; qualitative data underwent content analysis.

**Results:**

A total of 172 participants were included, predominantly presenting with stress-related, depressive and anxiety disorders. High patient satisfaction was reported for IP, particularly in terms of ease of contact, patient involvement and respectful treatment. The IP team described that the triage process was challenging yet meaningful, relying heavily on an interprofessional team with strong clinical expertise and a person-centred approach. Improved integration with regular care could further enhance the service. The response rate among primary care clinicians was low, with responses indicating moderate acceptability.

**Conclusions:**

Telepsychiatry for triage delivered through a regional app was feasible, acceptable and may improve access to timely assessments. The findings also highlight important organizational and implementation challenges, particularly regarding integration with primary care, which should be addressed alongside evaluations of long-term outcomes, healthcare use and costs and policy barriers in future studies.

## Introduction

Telepsychiatry, delivering mental healthcare at a distance using digital technologies, has expanded steadily with COVID-19 accelerating adoption as telehealth shifted from supplemental to central in healthcare delivery.^1,2^ This shift has increased acceptance among both patients and providers, and a growing body of evidence suggests that telehealth can improve access and efficiency while achieving satisfaction and outcomes comparable to in-person care.^3,4^ Within this development, tele-triage systems have been shown to reduce unnecessary visits, enhance safety and guide patients to the appropriate level of care^5,6^ while pre-consultation digital history gathering tools can further support diagnostic accuracy in primary care.^7,8^ Yet important challenges remain, including technical barriers, limited trust, digital literacy, privacy concerns and insufficient coordination across levels of care.^9–11^ Such barriers are particularly evident in complex cases requiring interdisciplinary collaboration.^12^ Despite many trials and reviews, few studies have reported real-world implementation,^13^ highlighting the need for empirical case studies to guide best practice. Recent research shows that remote consultations are increasingly used in primary and mental healthcare, revealing both opportunities and challenges related to accessibility, engagement and quality of care.^14^

In Sweden, an official inquiry report highlighted the need for person-centred, integrated care with digital triage.^15^ About one-third of primary care patients present with psychiatric conditions that are managed within primary care, often without access to specialist psychiatric expertise,^16,17^ while limited psychiatric resources and poor continuity further delay treatment and worsen outcomes.^18,19^ The gap between primary care and specialist psychiatry has long been recognized, yet it persists.^20^ Collaborative Care Models (CCMs) were developed to address this gap by adding a behavioural care manager and consulting psychiatrist to the primary care team, combined with systematic outcome monitoring and stepped, person-centred care. CCMs have consistently improved outcomes for depression and anxiety and expanded access in underserved settings.^21,22^ While CCMs represent one pathway to stronger integration, fewer studies have examined digital solutions for triage and coordination.

In this context, Region Stockholm piloted Immediate Psychiatry (IP) in 2022. Delivered through the official regional healthcare app, IP emphasized security and trust while offering same-day video triage with mental health professionals. Sweden's high digital penetration reduced potential technical barriers.^23^ Supported by a multidisciplinary team across primary, secondary and emergency care, IP was designed as a low-threshold entry point to psychiatric assessment. This study evaluates its real-world implementation.

## Aims of the study

The aim of this mixed-methods pilot study was to implement and describe the Immediate Psychiatry (IP) tele-triage service, examine patient flow and characteristics and assess its feasibility, acceptability and user experiences. The findings are intended to inform researchers, policymakers and practitioners on the development of similar interventions to enhance access to mental healthcare. Specifically, we sought to address the following research questions, grouped into four domains:

1. Service use and flow
  - *How many patients used different stages of the IP service during the study period?*
  - *What subsequent level of care (primary versus specialist care) were recommended after triage?*
2. Patient characteristics
  - *What were the demographic characteristics of patients (e.g., age, gender, type of psychiatric problems)?*
  - *How did patients rate their psychiatric concerns in terms of symptom type, severity, onset and duration?*
  - *How did the IP team assess patients’ psychiatric symptoms and suicidality?*
3. Acceptability and feasibility
  - *How satisfied were patients with the IP service?*
  - *How did participating primary care centres rate the acceptability of IP?*
4. Team experiences
  - *How did the IP team experience their work with the intervention?*

## Method

### Study design, setting and ethical considerations

This pilot study was conducted at a psychiatric clinic affiliated with Karolinska Huddinge Hospital in collaboration with eight primary care centres in southern Stockholm. A mixed-methods design was used, giving equal priority to quantitative and qualitative data, which were collected in parallel and integrated at the interpretation stage. Data were collected between December 19, 2022, and June 30, 2023. The study was approved by the Swedish Ethical Review Authority (2022-03893-01), which waived the requirement for written informed consent. Participants received mandatory written information about the study in the app prior to completing the intake questionnaire and scheduling a consultation. Participation in the research component was voluntary, and all participants were informed of their right to opt out or withdraw at any time without consequences for their care. This consent procedure was explicitly approved by the Ethics Committee. Patients who completed the screening but did not proceed to booking or consultation were not contacted for follow-up, as a formal care relationship was not established prior to a completed consultation and this was outside the scope of the ethical approval.

## Recruitment

Information about the IP service was advertised on participating primary care web sites, posters, and in local newspapers and schools, and was also provided verbally by healthcare staff. All information was provided in Swedish. Patients accessed the service via Region Stockholm's healthcare app ‘Alltid Öppet’, which was widely used during the COVID-19 pandemic.

## Participants

Inclusion criteria were age 18 years or older and registration at one of the eight participating primary care centres. No exclusion criteria were applied, allowing inclusion of both subclinical and severe psychiatric concerns. Due to practical limitations regarding questionnaires and consultations, the service was primarily available for Swedish-speaking individuals.

## Intervention and procedure

### The IP service

**Figure 1.**
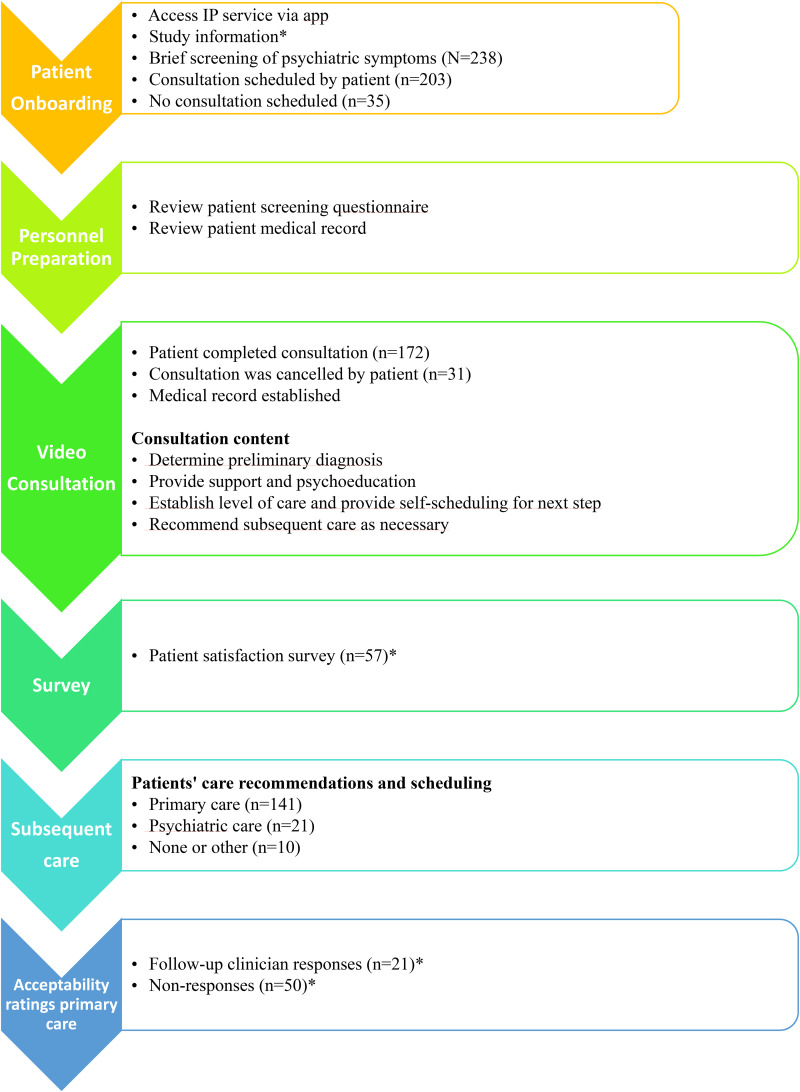
Flow of patients through the Immediate Psychiatry (IP) tele-triage service, illustrating progression from screening to completed consultation and subsequent referral outcomes. IP Service: Immediate Psychiatry Service.

The IP service was delivered via Region Stockholm's healthcare app ‘Alltid Öppet’. Logged-in patients completed a brief screening, scheduled a 45-min video consultation, and were invited to complete a post-consultation satisfaction survey (Figure 1). In this study, ‘triage’ refers to the initial evaluation aimed at determining the appropriate level of care and referral pathway, whereas ‘assessment’ denotes a more comprehensive clinical evaluation conducted during the consultation. The service was developed collaboratively by a multidisciplinary team (psychiatrist, psychologists and a psychiatric nurse) supported by a steering committee with representatives from primary and specialist care. Development followed an iterative process with regular feedback from stakeholders. A risk assessment was conducted and approved by the Chief Medical Officer, and continuous improvement was ensured through structured review meetings. A staff manual was published with the project report,^24^ covering protocols for consultations, suicide risk management, referrals and administrative routines.

* Activities related to voluntary participation study inclusion and data collection.

### Intake, screening, and patient satisfaction

At intake, patients received study information and completed a brief screening of psychiatric symptoms (Supplement A), including both structured and open-ended items. They then scheduled a video consultation with an IP mental health professional. Following the consultation, patients were invited to complete an anonymous satisfaction survey (Supplement B).

### Team organization and competencies

The IP team was multidisciplinary, comprising one psychiatrist, two psychologists, two psychiatric nurses, one therapist, two administrators and three alternating consulting psychiatrists. Two staff members also held a position at a collaborating primary care centre. Collectively, the team had broad experience from inpatient and outpatient psychiatry, medicine, addiction treatment, social psychiatry, services for persons with functional impairments, emergency and school-based care, suicide prevention and healthcare unit management.

### Consultation

Consultations were informed by patient brief screening self-reports and medical records. The team used a semi-structured interview guide originally developed at the hosting psychiatric clinic and adapted for IP (Supplement C) to obtain patient histories, complete triage and assess primary psychiatric concerns including symptoms, suicidality, substance use and domestic violence. A preliminary care plan was jointly developed with the patient. All consultations were conducted in Swedish.

### Clinical documentation

Consultations were documented in electronic medical records with preliminary diagnosis, care plan and planned or taken actions. Suicide risk was classified as minimal, moderate or high, and suicide attempts past 30 days and over the lifetime were recorded. Information on children in the household and exposure to violence or abuse was also noted.

### Subsequent care

Actions taken during or after consultation included provision of self-help guidance, referral to primary or specialist psychiatric care, referral to nongovernmental organizations or completion without referral. Decisions were based on the professional's assessment in dialogue with the patient and, when needed, in consultation with the team or a psychiatrist. Patients could self-schedule the follow-up appointments via the app.

## Data collection

### Patient characteristics and patient flow

Data on patient characteristics were collected through self-reported psychiatric concerns as well as preliminary diagnoses and actions initiated by the IP team, as indicated by information from the medical records. Patient flow was monitored through medical records.

### Brief screening of psychiatric concerns

A 9-item brief screening questionnaire (Supplement A) was developed by the IP steering committee, consisting of senior clinicians from primary and specialist psychiatric care, to support early information gathering prior to consultation. The purpose was to provide the mental health professional with an overview of areas to highlight during assessment and to explore the feasibility of a brief digital intake in a real-world telepsychiatry setting.

The questionnaire included both multiple-choice and open-ended items covering common psychiatric symptom domains (mood, anxiety, stress-related, obsessive–compulsive and psychotic symptoms), suicidality, psychiatric history and current psychotropic medication. A guiding principle in the development was that all items should be optional and that the number of items should be kept low in order to reduce patients’ cognitive load and minimize intake attrition. The screening questionnaire was not intended for diagnostic purposes and has not been formally psychometrically validated.

### Medical records

Data extracted from medical records included sex, age, preliminary diagnosis, suicide risk level, history of suicide attempts and actions planned or taken.

### Patient satisfaction

Patient satisfaction was measured with a nine-item survey on a 5-point Likert scale, covering access, communication, self-care advice, perceived effectiveness, staff competence and respect, involvement in decisions, appropriateness of follow-up care and likelihood of recommending the service. Items were adapted from validated Swedish national and regional patient satisfaction surveys.^25^ Although individual items were derived from established instruments, the questionnaire as used in this study has not been psychometrically validated as a composite measure in this specific context.

## IP team's experiences

### Interviews with the IP team

Experiences were explored through three 90-min retrospective semi-structured interviews conducted during the final month of the intervention with two psychiatric nurses and one therapist. At the time of data collection, three members of the IP team were available to participate in interviews, reflecting staff turnover and role changes during the pilot period. The fifteen open-ended questions (developed by project managers AP and UFO; Supplement D) covered roles, tasks, patient interaction, guiding principles and decision-making. Interviews were conducted by an independent psychologist in training with interview experience, the audio was recorded and transcribed using Whisper. All AI-generated transcripts were manually reviewed against the original audio recordings to correct transcription errors, including potential misinterpretations of clinical terminology, prior to analysis. The purpose was to understand clinical practices and support refinement of the IP model.

### Clinician acceptability at primary care centres

Clinicians at primary care centres receiving IP referrals were invited by email to complete the four-item Acceptability of Intervention Measure,^26^ rated on a 5-point Likert scale covering approval, appeal, personal liking and welcoming of the service.

## Data analysis

### Statistics

Descriptive statistics include group and category percentages, counts, means and standard deviations. Fisher's exact test was applied to analyse associations, and the Mann–Whitney U test to compare distributions. Questionnaire data are reported with means and standard deviations. Missing data for one AIM item were imputed using the mean of the other items in the same record.

### Qualitative content analysis

Qualitative content analysis was used to interpret interview data, following established frameworks.^27,28^ Transcripts were first reviewed and manually summarized for familiarity, then coded and synthesized into central themes. This method was chosen for its suitability in exploring complex, context-specific phenomena and its flexibility compared with other text analysis techniques. Analyses were initially conducted by AP and UFO, with SS assisting in later stages. AP and UFO were directly involved in the IP project, while SS contributed to scientific analysis and writing. All three authors are clinical psychologists with experience in primary care and psychiatry; UFO also works as a healthcare business developer within Region Stockholm. Two of the authors had insider roles in the IP service during the study period. Reflexivity was addressed through ongoing discussions within the research team, transparent documentation of analytic decisions and involvement of an additional author without direct operational responsibility in later stages of analysis and interpretation.

## Results

### Service use and flow

Figure 1 shows participant flow. In total, 238 patients initiated contact with IP via the app and completed the brief screening. Of these, 203 (85.3%) scheduled an appointment and 35 (14.7%) did not. Among those scheduled, 172 (84.7%) attended a consultation and were included in the study. After triage, 141 (82.0%) were recommended follow-up in primary care, 21 (12.2%) in specialist psychiatric care and 10 (5.8%) were referred to other alternatives, including services outside healthcare.

### Patient characteristics

Patient characteristics are presented in Table 1 and brief screening responses in Table 2. One patient withdrew consent for research after the consultation and was excluded, and all analyses are therefore based on *n* = 172. We conducted a sensitivity analysis excluding the ‘*Other*’ group (n = 10) which did not affect the results materially.

**Table 1.** Characteristics of patients receiving consultation, distribution of their preliminary diagnoses and assessed level of care.

Variables		Total sample (n = 172)	Referred to primary care (n = 141)	Referred to psychiatric care (n = 21)	Other^a^ (n = 10)	Primary vs psychiatric care^b^, p
Age in years, M (SD)	All	34.5 (12.5)	35.6 (12.6)	28.4 (11.1)	31.2 (10.9)	.003**
	Women	34.6 (13.4)				.603
	Men	34.2 (10.5)				
Sex, % (n)	Women	67 (116)				
	Men	33 (56)				
Preliminary diagnosis, % (n)	STR	25.6 (44)	30.5 (43)	0.0 (0)	10.0 (1)	.001**
	DEP	23.8 (41)	23.4 (33)	28.6 (6)	20.0 (2)	.592
	ANX	22.1 (38)	24.8 (35)	9.5 (2)	10.0 (1)	.165
	PD	9.9 (17)	7.1 (10)	23.8 (5)	20.0 (2)	.028*
	ADHD	3.5 (6)	2.8 (4)	4.8 (1)	10.0 (1)	.505
	OCD	2.9 (5)	2.1 (3)	9.5 (2)	0.0 (0)	.126
	PTSD	2.9 (5)	2.1 (3)	9.5 (2)	0.0 (0)	.126
	ASD	1.7 (3)	0.0 (0)	9.5 (2)	10.0 (1)	.016*
	SUD	0.6 (1)	0.0 (0)	4.8 (1)	0.0 (0)	.130
	Other	5.2 (9)	5.0 (7)	0.0 (0)	20.0 (2)	.596
	Missing	1.7 (3)	2.1 (3)	0.0 (0)	0.0 (0)	1.000
Suicide risk, % (n)	Minimal	52.3 (90)	58.2 (82)	28.6 (6)	20.0 (2)	.017*
	Moderate	45.3 (78)	40.4 (57)	61.9 (13)	80.0 (8)	.097
	High	1.2 (2)	0.0 (0)	9.5 (2)	0.0 (0)	.016*
	Missing	1.2 (2)	1.4 (2)	0.0 (0)	0.0 (0)	1.000
Previous suicide attempts, n (%)	Yes	7.0 (12)	5.0 (7)	23.8 (5)	0.0 (0)	.010**
	No	49.4 (85)	50.4 (71)	38.1 (8)	60.0 (6)	.353
	Missing	43.6 (75)	44.7 (63)	38.1 (8)	40.0 (4)	.642

STR: Stress-related Disorder, DEP: Depression Disorder, ANX: Anxiety Disorder, PD: Personality Disorder, ADHD: Attention-Deficit Hyperactivity Disorder, PTSD: Post-Traumatic Stress Disorder, OCD: Obsessive-Compulsive Disorder, ASD: Autism Spectrum Disorder, SUD: Substance Use Disorder, Other: No or Other Disorder.

a No referral or recommended contact outside healthcare.

b Mann-Whitney for values M (SD) and Fishers Exact for values n (%). Comparison between patients referred to primary and psychiatric care.

**Table 2.** Brief screening of psychiatric concerns by triaged level of care.

**Item**	**Total, % (n)**	**Primary care, % (n)**	**Psychiatric care, % (n)**	**Other^a^, % (n)**	**Primary vs psychiatric care^b^, p**
I am seeking help because …					
Something has happened that consumes my thoughts and makes me feel bad.	60.5 (104)	61.0 (86)	52.4 (11)	70.0 (7)	.481
I mostly feel sad and depressed. I rarely, if ever, feel joy or desire.	53.5 (92)	53.9 (76)	61.9 (13)	30.0 (3)	.639
I feel nervous, anxious, afraid, or experience panic. It interferes with my daily life.	58.1 (100)	57.4 (81)	57.1 (12)	70.0 (7)	1.000
I have intrusive and uncomfortable thoughts, or I believe things that are untrue.	29.7 (51)	28.4 (40)	28.6 (6)	50.0 (5)	1.000
I struggle with education/work, daily tasks, or relationships.	51.7 (89)	51.1 (72)	61.9 (13)	40.0 (4)	.483
None of the above.	20.3 (35)	17.0 (24)	42.9 (9)	20.0 (2)	.016*
Of which only: ‘None of the above’	4.1 (7)	3.5 (5)	4.8 (1)	10.0 (1)	.571
The issue I am seeking help for …					
Started after a specific event, or during a stressful period. I was well before.	50.0 (86)	53.2 (75)	33.3 (7)	40.0 (4)	.105
Has troubled me intermittently. During other periods, I've been well.	51.2 (88)	52.5 (74)	38.1 (8)	60.0 (6)	.249
Has troubled me for a long time, perhaps as long as I can remember.	43.0 (74)	38.3 (54)	71.4 (15)	50.0 (5)	.008**
Has started or continued in some other way.	11.0 (19)	7.8 (11)	23.8 (5)	30.0 (3)	.038*
How much does your issue impact your daily life and relationships?					
Very much	61.0 (105)	58.9 (83)	66.7 (14)	80.0 (8)	.635
Quite a bit	36.0 (62)	37.6 (53)	33.3 (7)	20.0 (2)	.811
A little	2.9 (5)	3.5 (5)	0.0 (0)	0.0 (0)	1.000
Almost not at all	0.0 (0)	0.0 (0)	0.0 (0)	0.0 (0)	1.000
Do you have thoughts of harming yourself or not wanting to live?					
Yes	33.1 (57)	29.8 (42)	52.4 (11)	40.0 (4)	.048*
Have you previously been in contact with any mental health services for your issues?					
Yes	60.5 (104)	56.7 (80)	76.2 (16)	80.0 (8)	.102
Have had any previous psychiatric diagnoses?					
Yes	41.9 (72)	39.0 (55)	57.1 (12)	50.0 (5)	.154
Are you currently taking medication for mental health issues?					
Yes	23.3 (40)	19.1 (27)	42.9 (9)	40.0 (4)	.023*

a No referral or recommended contact outside healthcare.

b Fishers Exact for values n (%). Comparison between patients referred to primary and psychiatric care.

### Preliminary diagnosis

Patients’ preliminary diagnoses and subsequent care are reported in Table 1. The most common diagnoses were stress-related, depressive and anxiety disorders, followed by personality disorders; other diagnostic groups were less frequent. Referral pathways differed: no patients with stress-related disorders were referred to specialist care, no patients with autism spectrum disorders were referred to primary care, and patients with personality disorders were more often referred to specialist care.

### Suicidality

Most patients were assessed as having minimal suicide risk (52.3%), 45.3% as moderate and 1.2% as high. Low-risk patients were predominantly referred to primary care, whereas all high-risk patients were referred to specialist care. Among consultations with available data (56.4%), 7.0% reported previous suicide attempts, and these patients were more frequently referred to specialist care.

### Patient ratings

Brief screening responses (Table 2) most often concerned overwhelming thoughts, anxiety, and depressive symptoms and about half attributed onset to a specific stressful event. Most described intermittent problems, while a minority reported long-standing, likely chronic conditions. Symptom impact was high: nearly two-thirds selected the highest impairment level for daily life, and one-third the second highest. Over half of the patients had previous contact with mental healthcare and a minority reported self-harm thoughts or a wish not to live. Differences between referral groups were small overall, but patients referred to specialist care more often reported long-standing symptoms, self-harm thoughts and current psychiatric medication use, than patients referred to primary care. On average, patients endorsed 2.53 (SD = 1.25) of the six listed symptoms, a Mann–Whitney U test showed no significant group difference (p = 0.704).

## Acceptability and feasibility

### Patient satisfaction

Patient satisfaction with the consultation was high (Table 3), with all item means above 4 on a 1–5 scale. The highest scores concerned timely access (M = 4.96, SD = 0.27), respectful and considerate treatment (M = 4.95, SD = 0.23), and involvement in care decisions (M = 4.88, SD = 0.38).

**Table 3.** Patient satisfaction survey (n = 57).

Item	M (SD)
Were you able to contact IP within a reasonable time?	4.96 (0.27)
Did you feel comfortable telling the staff how you are feeling?	4.72 (0.45)
Are you satisfied with the self-care advice you received?	4.36 (0.96)
Do you feel that you have been helped by the consultation?	4.33 (1.01)
Did you find the staff knowledgeable?	4.75 (0.61)
Did you feel that you were treated with respect and in a considerate manner?	4.95 (0.23)
Did the staff involve you in the decisions about your care/treatment?	4.88 (0.38)
If offered a follow up; were you satisfied with choice of receiving clinic?	4.57 (0.76)
Would you recommend contacting IP to someone in a similar situation?	4.80 (0.55)

M = mean; SD = standard deviation.

### Clinician acceptability in primary care

The clinicians’ acceptability of the IP is summarized in Table 4. The survey was sent to 71 clinicians, and 21 (29.6%) responded. Overall, clinicians reported moderate acceptability, with mean scores between 3.42 and 3.53 (SD = 1.54–1.66) across all four items (scale 1–5).

**Table 4.** Acceptability ratings from primary care clinicians (n = 21).

Item	M (SD)
IP meets my approval.	3.50 (1.54)
IP is appealing to me.	3.42 (1.66)
I like IP.	3.42 (1.63)
I welcome IP.	3.53 (1.57)

M = mean; SD = standard deviation.

### Team experiences

The IP team's experiences with their work are described in the following categories:

1. Patient-centred care and effective communication strategies

The IP team emphasized their commitment to holistic and patient-centred care, recognizing each patient as a unique individual with specific needs and circumstances. They described prioritizing active listening over relying on medical records, in order to gain a more nuanced understanding of the patient's situation:

It is more important to truly listen to the patient and understand their experiences than to just go through their medical records. By listening actively, we gain a more nuanced understanding of the patient's situation. (Nurse 1)

The core values of respect and equity were highlighted, ensuring that care was fair and tailored for the individual. Involving patients in decisions was said to foster empowerment and engagement, while validating patients’ feelings was seen as crucial in challenging conversations. By creating a supportive and empathic environment, the team aimed to help patients experience being understood and accepted, which they believed was essential for building trust and a therapeutic alliance.

Attuned communication was described as a cornerstone of the work. Team members adapted their language and interaction to the patient's age, comprehension and cultural background to ensure meaningful dialogue. Allowing patients, particularly those with trauma, to express themselves in their own way was reported to create a sense of being heard and understood. Consultations typically followed a structured process: the patient told their story, the clinician asked follow-up questions and the session concluded with a summary and plan. One nurse described this approach:

At the beginning of the conversation, I introduce myself and outline the timeframe. I usually divide the conversation into three parts: one where you can tell me more about yourself, then I will have follow-up questions, and at the end, we will do a summary and plan for the future. I say these things so that the patient has some idea of what will happen, even if they don’t know exactly what questions I will ask, they still have a kind of agenda for the conversation. (Nurse 1)

Technical reliability was described as critical. Functional systems were necessary to avoid stress and to ensure smooth communication. An optimal physical environment was also important, with both the caregiver and patient in a quiet and distraction-free setting, something that was not always the case.

2. Interdisciplinary expertise and collaboration

The team highlighted the strength of their diverse expertise, which spanned psychiatric inpatient care, social psychiatry and addiction treatment. This breadth of experience was said to enable comprehensive assessments and care planning, where different perspectives were integrated for the patients’ benefit. Collaboration within the team was described as central and supported by secure communication tools such as video and chat, which facilitated both scheduled meetings and spontaneous discussion. These practices fostered cohesion and camaraderie, which the team regarded as essential for maintaining high-quality care.

In complex cases, particularly involving suicidality, the importance of interdisciplinary collaboration was emphasized. Team members noted that access to on-call consulting psychiatrists was invaluable for ensuring safe and well-informed decisions:

On the occasions when you need to consult with the on-call psychiatrist, it often concerns suicide. And it is very gratifying to have that consultation, to have that opportunity. (Therapist)

Several participants reflected on how their previous professional experiences supported their work in the IP service:

I worked in psychiatric inpatient care as a nurse. There, you also had the opportunity to meet all kinds of patients, psychotic, depressed, manic, suicidal, all types of patients. And then also this work with suicide prevention that I have done. I feel that I have benefited from it. Much of the work I did in the mobile emergency unit. Then, of course, you have the other background as well. That experience is always there. Especially with the initial assessment. There have been several suicide assessments. So, I feel that I have benefited from what I learned earlier. (Nurse 1)

At the same time, collaboration and integration across healthcare settings posed challenges. Boundaries between primary and specialist psychiatric care were not always clear, complicating patient pathways. While some of the eight collaborating primary care centres engaged well, others presented obstacles, particularly in securing appointments for patients assessed as suitable for primary care. These inconsistencies were described as notable hurdles to ensure seamless and timely care.

3. Comprehensive assessment and care planning

The team strived to conduct thorough and comprehensive assessments of each patient. They described giving special attention to patients with suicidal thoughts or complex mental health issues and using detailed follow-up questions to gain a deeper understanding of their situation. Balancing the patients’ wishes with clinical assessments and consulting with colleagues and psychiatrists was said to ensure appropriate and efficient care planning. In consultations, self-care advice and referrals were often provided based on the comprehensive manual. This advice covered areas such as lifestyle, sleep, stress, diet and mindfulness, helping patients maintain their well-being.

When assessing and caring for suicidal patients, the team emphasized the importance of asking in-depth follow-up questions to obtain a complete picture of the patient's situation. Patients with suicidal thoughts were referred to psychiatric emergency units for in-person assessment and suicide prevention planning when it was unclear whether a referred service could offer the appropriate attention. Following the semi-structured assessment guide and gathering all necessary information was found to be crucial for ensuring appropriate care.

The IP service was described as having provided prompt, accurate and comprehensive assessments, serving as valuable support for both psychiatric services and primary care, as illustrated in the following quote:

I believe that to some extent, they have (patients with more severe conditions have discovered the IP services). A broader group has accessed these services through the app. Additionally, primary care centres have referred more complex or challenging cases to us. In situations where they are uncertain, they consider it better for someone with a broader perspective to assess the issues. (Nurse 2)

The team perceived that the extended assessment time improved the quality of initial evaluations.

We have more time to do so. And then we can ensure that the patient receives a timely follow-up. (Nurse 2)

4. Accessibility and innovation in service delivery

The team highlighted the accessibility of IP as a core strength, with short waiting times and flexible scheduling seen as highly valuable. Being able to meet patients in their own safe and familiar environments, typically at home, was described as reducing barriers to care and encouraging help-seeking among individuals who had not previously accessed mental healthcare. This was considered one of the service's most innovative features.

Continuous improvement of methods and practices was also emphasized. Scheduled team meetings provided a forum for evaluating challenges, refining procedures and sharing strategies. Several team members expressed a desire for more frequent consultations with psychiatrists to further strengthen clinical decision-making.

Flexibility during consultations was regarded as essential. Professionals described occasionally extending the 45-min sessions when needed, prioritizing completion of the assessment and establishing a therapeutic alliance:

But it is still better that I run over time … than that it doesn’t get finished, which I have done many times as well. But then, also, many patient meetings are about creating an alliance, and that is often something that happens over time, like when you meet a patient, and you meet them many times. But here, we must do it, and preferably do it all at once. And then it's about making the patient feel, exactly what I was mentioning earlier, that manner of interaction, it is very important. (Nurse 1)

Taken together, the accessibility, flexible scheduling and possibility for patients to engage from home were considered central to the service's success. At the same time, the team noted the importance of continued innovation, including closer integration with psychiatric consultation.

5. Ethical and practical considerations

Several ethical challenges were described in the tele-triage work. Situations where patients were not alone during video calls sometimes limited their ability to speak freely, raising concerns about privacy and autonomy.

The team also highlighted the importance of managing trust. Some patients had previous negative experiences of healthcare, which could affect their willingness to disclose sensitive information. In these situations, the professionals emphasized balancing professional boundaries with empathic engagement, while consistently following ethical guidelines to protect confidentiality.

Maintaining patients’ privacy and dignity was considered central to building trust and ensuring high-quality care. The team stressed that adherence to these principles was a prerequisite for delivering safe, equitable and respectful treatment in a digital format.

## Discussion

This study implemented and evaluated a telepsychiatric triage service integrated across primary and specialist care in Region Stockholm. A total of 172 patients were assessed, predominantly women presenting with anxiety, depressive and stress-related symptoms. Most patients were referred to primary care (82%), while 12% required specialist psychiatric follow-up. Patient satisfaction was high, whereas clinician acceptability in primary care was moderate. The IP team described improved access to patient-centred assessments, but identified integration with primary care as the main implementation challenge. The service managed patients across varying levels of suicide risk, demonstrating that digital triage can be applied within clinically complex populations.

### Patient characteristics and service utilization

The predominance of women and the symptom profile are consistent with findings from general^29^ and primary care populations.^30^ The age distribution suggests that app-based triage may be acceptable across adult age groups.^31^ The variation in symptoms, suicidality and psychosocial difficulties, together with the need for follow-up in primary care, illustrates the breadth of mental health needs encountered in routine care.^15,19^

### Telepsychiatry practice and patient experience

The findings highlight the importance of clinical competence and structured assessment, in line with prior research on remote consultations.^9,10^ High accessibility, short lead times, sufficient time for assessment and access to interprofessional expertise were described as central to perceived quality and patient satisfaction.^32^ Ethical and practical issues, including privacy during video consultations and maintaining trust, were also salient and should be considered in implementation.^33^ Patient satisfaction was very high, but the findings should be interpreted with caution due to voluntary participation in the survey (n = 57), which may introduce self-selection. Still, the results align with previous work showing that video-based consultations can support engagement and involvement in care.^33,34^

### Primary care acceptability and integration

Feedback from primary care clinicians was limited (21/71) and indicated moderate acceptability. The low response rate increases uncertainty and potential response bias; therefore, the results reflect only the views of responding clinicians, and the direction of any bias cannot be determined. The IP team also reported collaboration challenges with some centres, suggesting that implementation barriers may relate to organizational fit and local routines in addition to individual clinician attitudes.

The service introduced new cross-level workflows, including external booking into primary care schedules and short-notice follow-ups. While this may improve access for patients, it may also challenge established scheduling practices and role expectations in primary care. Given that most patients were directed to primary care after triage, successful implementation likely requires clear referral pathways, shared responsibilities and adequate capacity in receiving services. Telepsychiatry interventions appear more sustainable when embedded within established collaborative care structures,^35^ which may provide a clearer framework for coordination across care levels. Although cost-effectiveness was not evaluated, system-level impact will depend on organizational alignment and workflow integration.^36^ Digital triage may redistribute workload across care levels rather than reduce overall demand. Future research should therefore assess longer-term outcomes, healthcare use and economic sustainability.

### Generalizability

Generalizability may be limited by the narrow geographic setting and the Swedish context, including a publicly funded healthcare system, high digital literacy, and widespread use of the regional health app. In settings with different referral pathways or digital infrastructure, implementation challenges may differ. However, key elements – structured digital triage, video-based psychiatric assessment and interprofessional collaboration across care levels – may be transferable to other systems facing access constraints.

### Strengths and limitations

This study has several strengths. It represents a real-world implementation carried out within routine care settings and captures perspectives from patients, practitioners and collaborating primary care centres. The use of both quantitative and qualitative methods provides a comprehensive view of the intervention.

The service was offered only to Swedish-speaking patients during the pilot phase, which limits accessibility for non-Swedish-speaking populations. While this was a pragmatic constraint in the pilot, language adaptation and access to interpreters will be essential for equitable implementation of tele-triage services in routine care, particularly given the role of such services as early entry points to mental health care.

Substantial drop-off was observed between initial screening, booking and completed consultation. From one perspective, this reflects missing data, but from a feasibility perspective it represents important information regarding service use and flow across different stages of a digital triage service, where patients are free to discontinue the process at any point. As follow-up of patients who did not complete a consultation was not included in the ethical approval, information on individual reasons for non-attendance could not be collected. This limits conclusions regarding why some patients did not proceed beyond specific stages. However, based on information provided by patients who completed consultations, parallel care-seeking and mismatch between patient needs and available appointment times are plausible explanations.

Several questionnaires were pragmatically developed or adapted for this clinical context. The lack of formal psychometric validation limits conclusions about reliability and construct validity, and voluntary participation in the patient satisfaction survey may have introduced self-selection. Primary care clinician acceptability was based on a low response rate, which increases uncertainty and risk of response bias; the results reflect only responding clinicians, and the direction of potential bias cannot be determined.

Not all members of the IP team were interviewed, which may have reduced the breadth of perspectives represented. The qualitative findings should therefore be interpreted as providing context-specific insights into implementation experiences rather than claims of theoretical saturation. Furthermore, two of the authors involved in the qualitative analysis were closely integrated into the project, offering valuable insider knowledge but also a potential bias toward its benefits. Conversely, the remaining authors were less directly involved in the IP team but contributed extensive expertise in mental healthcare and digital interventions within psychiatry and primary care.

### Clinical implications

Delivering prompt and meaningful care that patients value requires experienced and competent staff, with the ability to consult effectively with both colleagues and physicians. Strengthening the integration of tele-triage within healthcare systems, particularly bridging the gap between primary and specialist psychiatric care, is essential. Effective integration can enhance accessibility, improve quality of care and support the coordination of services across different levels of care.

While efficient triage is a critical component of accessible and appropriate mental healthcare, it cannot compensate for insufficient treatment resources. Ensuring that primary care has the necessary capacity and resources to manage the influx of patients triaged from IP is therefore critical for the intervention's overall success. Such capacity not only benefits patient outcomes but also supports the healthcare system in providing comprehensive, cohesive and long-term sustainable care.

### Future research

Future studies should examine the long-term effects of tele-triage interventions such as IP on patient outcomes, healthcare resource utilization and cost-effectiveness. Larger randomized controlled trials are warranted to evaluate the broader impact of these interventions on healthcare systems. In addition, qualitative research exploring the perspectives of primary care providers could yield valuable insights into the practical challenges and facilitators of integrating tele-triage services into routine care.

## Conclusion

This pilot study demonstrates the feasibility of implementing single-session, video-based psychiatric triage and assessment consultations. The findings of high patient satisfaction and moderate clinical acceptability indicate that the intervention has potential as a standard entry point for mental health concerns across primary and secondary care. For sustainable implementation, integration into routine healthcare is essential, ideally through models that support collaboration and treatment coordination, such as the Collaborative Care Model (CCM), in order to enhance accessibility, continuity and patient outcomes.

## Supplemental Material

sj-pdf-1-dhj-10.1177_20552076261429684 - Supplemental material for Telepsychiatry for mental health triage: A mixed-methods pilot study via a regional health app in Sweden Supplemental material, sj-pdf-1-dhj-10.1177_20552076261429684 for Telepsychiatry for mental health triage: A mixed-methods pilot study via a regional health app in Sweden by Andreas Påhlsson-Notini, Sigrid Salomonsson, Elin Lindsäter, Sara Arvas, Ulla Forsbeck Olsson, Oscar Norbeck, Maria Bragesjö, Viktor Kaldo and Lina Martinsson in DIGITAL HEALTH

sj-pdf-2-dhj-10.1177_20552076261429684 - Supplemental material for Telepsychiatry for mental health triage: A mixed-methods pilot study via a regional health app in Sweden Supplemental material, sj-pdf-2-dhj-10.1177_20552076261429684 for Telepsychiatry for mental health triage: A mixed-methods pilot study via a regional health app in Sweden by Andreas Påhlsson-Notini, Sigrid Salomonsson, Elin Lindsäter, Sara Arvas, Ulla Forsbeck Olsson, Oscar Norbeck, Maria Bragesjö, Viktor Kaldo and Lina Martinsson in DIGITAL HEALTH

sj-pdf-3-dhj-10.1177_20552076261429684 - Supplemental material for Telepsychiatry for mental health triage: A mixed-methods pilot study via a regional health app in Sweden Supplemental material, sj-pdf-3-dhj-10.1177_20552076261429684 for Telepsychiatry for mental health triage: A mixed-methods pilot study via a regional health app in Sweden by Andreas Påhlsson-Notini, Sigrid Salomonsson, Elin Lindsäter, Sara Arvas, Ulla Forsbeck Olsson, Oscar Norbeck, Maria Bragesjö, Viktor Kaldo and Lina Martinsson in DIGITAL HEALTH

sj-pdf-4-dhj-10.1177_20552076261429684 - Supplemental material for Telepsychiatry for mental health triage: A mixed-methods pilot study via a regional health app in Sweden Supplemental material, sj-pdf-4-dhj-10.1177_20552076261429684 for Telepsychiatry for mental health triage: A mixed-methods pilot study via a regional health app in Sweden by Andreas Påhlsson-Notini, Sigrid Salomonsson, Elin Lindsäter, Sara Arvas, Ulla Forsbeck Olsson, Oscar Norbeck, Maria Bragesjö, Viktor Kaldo and Lina Martinsson in DIGITAL HEALTH

sj-pdf-5-dhj-10.1177_20552076261429684 - Supplemental material for Telepsychiatry for mental health triage: A mixed-methods pilot study via a regional health app in Sweden Supplemental material, sj-pdf-5-dhj-10.1177_20552076261429684 for Telepsychiatry for mental health triage: A mixed-methods pilot study via a regional health app in Sweden by Andreas Påhlsson-Notini, Sigrid Salomonsson, Elin Lindsäter, Sara Arvas, Ulla Forsbeck Olsson, Oscar Norbeck, Maria Bragesjö, Viktor Kaldo and Lina Martinsson in DIGITAL HEALTH

## List of abbreviations

IP: immediate psychiatry
AIM: acceptability of the intervention measure
CCM: collaborative care
