# Supplementary material for: Telepsychiatry for mental health triage: A mixed-methods pilot study via a regional health app in Sweden
Source: Digit Health. 2026 Mar 10;12:20552076261429684. doi: 10.1177/20552076261429684 (PMC12979915; doi:10.1177/20552076261429684)
Supplement: sj-pdf-1-dhj-10.1177_20552076261429684 - Supplemental material for Telepsychiatry for mental health triage: A mixed-methods pilot study via a regional health app in Sweden [file sj-pdf-1-dhj-10.1177_20552076261429684.pdf]

Supplement A) Brief Screening of Psychiatric Concerns

Question 1 – I am seeking help because…
...something has happened that consumes my thoughts and makes me feel bad.
...I mostly feel sad and depressed. I rarely, if ever, feel joy or desire.
...I feel nervous, anxious, afraid, or experience panic. This interferes with my daily life.
...I struggle with education/work, daily tasks, or relationships.
...I have intrusive and uncomfortable thoughts, or I believe things that are untrue.
None of the above.
(multiple choice)

Question–2 - The issue I am seeking help for...
...started after a specific event, or during a stressful period. I was well before.
...has troubled me intermittently. During other periods' I've been well.
...has troubled me for a long time, perhaps as long as I can remember.
...has started or continued in some other way.
(multiple choice)

Question–3 - How much does your issue impact your daily life and relationships?
Very much
Quite a bit
A little
Almost not at all
(Single choice)

Question–4 - Do you have thoughts of harming yourself or not wanting to live?
Yes
No
(Single choice)

Question–5 - Have you previously been in contact with any mental health services for your issues?
Yes
No
(Single choice)

Question–6 - Do you consent to us accessing your medical records from other healthcare providers?
(For optimal assistance, we require access to your medical records.)
Yes
No
(Single choice)

Question–7 - Do you have had any previous psychiatric diagnoses?
Yes
No
(Single choice)

Question–8 - Are you currently taking medication for mental health issues?
Yes
No
(Single choice)

Question–9 - Please describe your issues and the help you seek in your own words.
(Free text)
