# Supplementary material for: Telepsychiatry for mental health triage: A mixed-methods pilot study via a regional health app in Sweden
Source: Digit Health. 2026 Mar 10;12:20552076261429684. doi: 10.1177/20552076261429684 (PMC12979915; doi:10.1177/20552076261429684)
Supplement: sj-pdf-2-dhj-10.1177_20552076261429684 - Supplemental material for Telepsychiatry for mental health triage: A mixed-methods pilot study via a regional health app in Sweden [file sj-pdf-2-dhj-10.1177_20552076261429684.pdf]

Supplement B) Questionnaire - Patient Satisfaction Survey

1 - Were you able to get in touch with IP within a reasonable time?
2 - Did you feel comfortable telling the staff how you are feeling?
3 - Are you satisfied with the self-care advice you received?
4 - Do you feel that you have been helped by the consultation?
5 - Did you find the staff knowledgeable?
6 - Did you feel you were treated with respect and in a considerate manner?
7 - Did the staff involve you in decisions about your care/treatment?
8 - If offered a follow up; were you satisfied with choice of receiving clinic?
9 - Would you recommend contacting IP to someone in a similar situation?

(Likert scale, 1 – 5, “No, not at all”- “Yes, completely”)
