# Supplementary material for: Telepsychiatry for mental health triage: A mixed-methods pilot study via a regional health app in Sweden
Source: Digit Health. 2026 Mar 10;12:20552076261429684. doi: 10.1177/20552076261429684 (PMC12979915; doi:10.1177/20552076261429684)
Supplement: sj-pdf-3-dhj-10.1177_20552076261429684 - Supplemental material for Telepsychiatry for mental health triage: A mixed-methods pilot study via a regional health app in Sweden [file sj-pdf-3-dhj-10.1177_20552076261429684.pdf]

IP 45	**Immediate Psychiatry** Consultation and assessment	**Social security number:** Version 0.1 220319 ENG	
		**Name:**	
		**Date:**	**Time:**

**Form of contact:** ☐ Video consultation	**Others present in consultation:** (name, relation)	**Interpreter in consultation:** (name, language)

Basic consultation

Introduction: Hello and welcome. My name is [name], and I am [profession]. We have around 45 minutes, during which we will explore what kind of help you need and how best to receive it. Immediate Psychiatry does not offer treatment, but we can and will refer you if needed. Before we begin, are you alone in the room? It's essential that we can speak confidentially.

Resons for helpseeking: What are you seeking help for? (What are your three main issues?). What do you think is the cause of these issues, and how do you think they could be improved? Do you have any family history of these issues? **If yes, explore further.**

(Blue = there are related questions in the app)

1: ______

2: ______

3: ______

Function: How does this affect you? Are you usually able to do what you need to do, such as earn a living, take care of everyday tasks and maintaining social connections?

No impact. Impact on work/school, everyday tasks, social connections, other.

Progression: How long has it been like this? Were things completely well before this? Has it been the same the whole time, or has is fluctuated? Have there been periods when you felt completely well? Was there any particular event or circumstance in your life that you think might have triggered your issues at that time?

Stressors : Do you often feel burdened by something around you? Has something in particular happened that affects you and that you find it difficult to deal with?

Social setting: What is your living situation? What do you spend your days doing? Do you have a partner, family, and friends? How is your financial situation? Do you have children? **If so, explore their ages and whether the patient has any concerns about a child being at risk.**

Living alone, with partner (+children), with children, with primary family, student housing. Studying, working full-time/part-time, on sick leave, job-seeking, on parental leave, retired. No children, one child, two children, three children… Many friends, limited social network, very limited social contacts. Good, strained, very strained finances.

Violence, abuse and threats: Have you ever been subjected to physical, psychological or sexual violence? Have you ever been violent yourself, or had violent thoughts?

Not subjected to violence. Subjected to physical, psychological, sexual violence. 1–2 occasions, repeated occasions. Never been violent. Been physically, psychologically, sexually violent. 1–2 occasions, repeated occasions.

Alcohol and drugs: Do you drink alcohol? When did you last drink? How much did you drink then? And when was the last time before that? Do you use any non-prescription drugs? (Use standard units))

Do not drink alcohol. Drinks alcohol + number of standard units in the last 30 days. Does not use drugs. Uses drugs occasionally, regularly. Types of drugs used: cannabis, cocaine, amphetamines, heroin, hallucinogens, non-prescription drug.

Suicidality and self-harm: How do you feel about your life at the moment? Have you ever wished you were no longer alive, or had thoughts about not waking up? Have you had any thoughts of ending your life? If so, please tell me more about how often these thoughts occur and how intense they feel. Have you ever engaged in self-harm? **If thoughts are present, examine severity, frequency and duration.** Do you ever harm yourself in any way? **If yes, investigate frequency, method, injuries, and any need for treatment of those injuries.**

No suicidal thoughts. Has thoughts of death, suicidal thoughts, suicide plans.

Physical health: Do you have any physical ailments or illnesses?

Current and previous care: What have you done so far to improve your condition? Have you previously been in contact with healthcare for your issues? Do you use any prescription drugs? **Investigate the nature, extent and effect of previous treatment.**

No previous healthcare contact for the problem, contact in primary care, contact in psychiatry. No previous psychological treatment. Previous talk support, CBT, PDT , other therapy. No medicines. Medication as needed, daily use.

Chronic conditions: ADHD, Autism spectrum disorder, intellectual disability

Have you been diagnosed with any of these conditions? Have there ever been concerns or suspicions about these conditions, such as from parents or teachers? How did you find your experience in school? **Investigate difficulties, grades, peer relationships, any support measures.**

No diagnosis. Has been investigated but not diagnosed. Diagnosed with ADHD, Asperger's syndrome, other autism spectrum disorder, intellectual disability.

Suicidality Continued

You mentioned having thoughts about ending your life. Have you considered how you would do it? (If yes, explore method, timing, tools, preparations) **Examine risk factors (see below).** Do you think you could take your own life? How likely is it that you would take your own life in the next month/week? What could stop you from doing so? **Examin protective factors (see below). Conduct a risk assessment.**

Risk Factors: Male, age >65, prior attempts, serious self-harm, depression, bipolar disorder, schizophrenia, personality disorder, severe anxiety, substance abuse, family history, LGBTQ, unaccompanied migrant, rejected asylum, bullying, perpetrator of violence, victim of violence, chronic illness/pain, life crisis, loneliness, severe vulnerability/trauma, hopelessness. Protective Factors: Seeking help, social network, optimism about the future, belief in one's ability to change, healthy habits, good contact with healthcare. Risk Assessment: Minimal, moderate, high, very high.

Depression, Bipolar Disorder

You mentioned feeling down. How are things with… **(check depressive symptoms below)**? Have you felt this way before? **If yes, explore frequency, duration, seasonality.** Have you ever had periods where you felt overly energetic or hyperactive? **If yes, explore degree, duration, possibly implusive behaviour.**

Symptoms: Reduced ability to feel joy, low energy, difficulty concentrating, reduced initiative, decreased/increased appetite, decreased/increased sleep, hopelessness, negative thoughts/self-blame. Number of prior depressive episodes. Overactivity, reduced need for sleep, racing thoughts, difficulty focusing, irritability, reckless behavior.

Anxiety and worry (Social Anxiety, GAD, specific phobias, health anxiety, panic disorder, OCD, PTSD)

You mentioned feeling anxious or worried. In what situations do you feel this way?? **Explore triggers (see below).** Do you ever experience anxiety that turns into a panic attack **Explore panic attack symptoms**. How do you manage your anxiety? Is there anything you avoid doing to prevent anxiety? How does this affect your daily life?

Triggers: Social situations, specific situations/stimuli (e.g., heights, animals), health symptoms or thoughts, everyday situations or thoughts, situations you can't escape from, nightmares/flashbacks, compulsive thoughts, other. Number or frequency of panic attacks. Avoidance: None, external/internal, compulsions. Functional Impairment: None, mild, moderate, severe.

Sleep Issues

You mentioned sleep problems. Can you tell me more about what they look like? How many nights per week do you have trouble sleeping on average? How many hours of sleep do you get on average? What have you tried so far to improve your sleep? How do sleep problems affect your daily life?

Symptoms: Difficulty falling asleep, waking up at night, waking up too early. Number of nights/week. Average sleep/night. Regular/irregular sleep times. Shift work yes/no. Tried/not tried tips on good sleep hygiene. No/certain/great/very great disability in everyday life. Motivated/not motivated for sleep restriction.

Risk Use/Substance Use/Dependence

I'd like to return to the topic of alcohol/drugs/other and talk more about how it affects your daily life, such as relationships, finances, work. Have you ever done something under the influence that had negative consequences, such as being in an accident or driving while intoxicated? How motivated are you right now to make a change, on a scale of 0–100%?

Risk Use: >14 standard drinks/week for men, >9 for women. Binge Drinking: At least 5 standard drinks per occasion for men, at least 4 for women. Abuse, addiction to alcohol, drugs, medication, gambling, other. Tolerance, withdrawal, preoccupation, loss of control, harmful behaviors. Motivated/Not motivated for change.

Eating disorders/disordered eating behaviour

You mentioned sometimes avoiding food or eating large amounts, or making yourself vomit afterward. Can you tell me more about this? How often does this happen? Do you find yourself preoccupied with thoughts about eating or not eating? If you think you've eaten too much, do you do anything afterward to make yourself feel better? Have you noticed changes in your weight because of this? How many kg approximately? Do you have any physical symptoms because of this? **For women: Ask about missed/irregular periods.** How does this affect your daily life? **ATTENTION! Distinguish between binge eating and overeating.**

Compensatory behaviors: Vomiting, laxatives, exercise, restricting food intake, other. Weight increased/decreased … kg since… Menstrual cycle: Regular, irregular, missed.

Closing

s there anything else that you think I should know? **Summarize the key points from the conversation. Describe available treatment options.** Do you have any preferences regarding these options? A**gree on the next steps, and inform about what will happen going forward, such as initiating appointment scheduling with a relevant unit. Encourage scheduling an appointment as soon as possible and to contact Immediate Psychiatry via chat if there are booking issues.**
