# Supplementary material for: Telepsychiatry for mental health triage: A mixed-methods pilot study via a regional health app in Sweden
Source: Digit Health. 2026 Mar 10;12:20552076261429684. doi: 10.1177/20552076261429684 (PMC12979915; doi:10.1177/20552076261429684)
Supplement: sj-pdf-4-dhj-10.1177_20552076261429684 - Supplemental material for Telepsychiatry for mental health triage: A mixed-methods pilot study via a regional health app in Sweden [file sj-pdf-4-dhj-10.1177_20552076261429684.pdf]

Supplement D) Interview Guide – Retrospective Interview with clinicians in the IP Team.

Introduction

Thank you for taking the time to participate in this interview. The objective is to explore and understand your role as a healthcare professional in your daily work. Questions will focus on your role, tasks, methodologies, and experiences. Your input is crucial for enhancement and development of the services provided.

Questions

1. Can you describe your professional role and primary tasks?
   • Discuss your background and tenure.
2. What types of conversations and cases do you most commonly handle?
   • Provide examples of typical patient cases and your approach to managing them.
3. How do you adapt your interactions and methods to accommodate diverse patient needs and conditions?
   • Cite examples where you've adjusted based on patient query, age, cultural background, diagnosis, or other influential factors.
4. What are the core principles or values guiding your work?
   • Discuss how you adhere to these principles and values in your patient interactions.
5. What methods and techniques do you employ to facilitate effective and supportive communication with patients?
   • Illustrate how you create a secure and open atmosphere during conversations.
6. When conducting video calls with patients, what aspects do you consider most vital for ensuring high-quality care and interaction?
   • Offer examples of how you ensure optimal care and support during video consultations.
7. How do you handle challenging conversations, such as when a patient is highly anxious or distressed?
   • Outline strategies and techniques you employ for handling such situations and best supporting the patient.
8. How do you and your colleagues make decisions concerning patient care plans, and what factors are considered?
   • Explain your decision-making process for interventions and factors involved.
9. What self-care advice and materials do you use when patients don't require further medical interaction?
   • Cite examples of such resources and how you utilize them in your assessments.
10. Are there any resources or materials you wish you had access to for enhancing patient care?
   • Discuss potential materials or resources beneficial to both you and the patients.
11. How do you collaborate with colleagues to ensure the best possible care for patients?
   • Describe your communication and collaboration around patient cases, and how you learn from each other.
12. How do you manage ethical questions and dilemmas arising in your work?
   • Provide examples of ethical dilemmas you've encountered and your approach to resolving them.
13. What challenges and success factors have you encountered in your work?
   • Share examples of successes and challenges, along with how you managed them.
14. Is there anything you wish to change or improve in your workflow and patient interactions?
   • Discuss possible changes or improvements beneficial to both you and the patients.
15. How are you involved in methodological development, and how do you have an opportunity to influence this process?
   • Describe your contributions to evolving and enhancing methodologies and how your input is valued.

Conclusion

Thank you for your participation and insights into your work! Your involvement is crucial for our ongoing improvement efforts. If you have further comments or questions, please feel free to share them now.
