# Supplementary material for: Telepsychiatry for mental health triage: A mixed-methods pilot study via a regional health app in Sweden
Source: Digit Health. 2026 Mar 10;12:20552076261429684. doi: 10.1177/20552076261429684 (PMC12979915; doi:10.1177/20552076261429684)
Supplement: sj-pdf-5-dhj-10.1177_20552076261429684 - Supplemental material for Telepsychiatry for mental health triage: A mixed-methods pilot study via a regional health app in Sweden [file sj-pdf-5-dhj-10.1177_20552076261429684.pdf]

**GRAMMS Criterion**	**Location in Manuscript**
**1. Justification for using a mixed methods approach to the research question**	Methods – Study design
**2. Description of the design in terms of purpose, priority, and sequence of methods**	Methods – Study design
**3. Description of each method in terms of sampling, data collection, and analysis**	Methods – Participants; Data collection; Data analysis (Quantitative and Qualitative)
**4. Description of where integration occurred, how it occurred, and who participated in it**	Methods – Study design; Data analysis; Discussion
**5. Description of any limitations of one method associated with the presence of the other method**	Discussion – Strengths and Limitations
**6. Description of insights gained from mixing or integrating methods**	Discussion
